# Advanced Oxidation Protein Product Promotes Oxidative Accentuation in Renal Epithelial Cells via the Soluble (Pro)renin Receptor-Mediated Intrarenal Renin-Angiotensin System and Nox4-H_2_O_2_ Signaling

**DOI:** 10.1155/2021/5710440

**Published:** 2021-11-26

**Authors:** Kai Xue, Yurong Wang, Yan Wang, Hui Fang

**Affiliations:** Key Laboratory of Applied Pharmacology in Universities of Shandong, Department of Pharmacology, School of Pharmacy, Weifang Medical University, Weifang, 261053 Shandong, China

## Abstract

Full-length (pro)renin receptor (fPRR), a research hotspot of the renin-angiotensin system (RAS), plays a serious role in kidney injury. However, the relationship between fPRR and advanced oxidation protein product (AOPP) remains largely unexplored. This study was aimed at exploring the effect of fPRR, especially its 28 kDa soluble form called soluble PRR (sPRR), in AOPP-induced oxidative stress in HK-2 cells, a renal proximal tubular epithelial cell line. Incubation of HK-2 cells with 100 *μ*g/ml AOPP resulted in significant upregulation of fPRR expression and caused an approximately fourfold increase in medium sPRR secretion. However, unmodified albumin did not demonstrate the same effects under the same concentration. Treatment of HK-2 cells with the site-1 protease (S1P) inhibitor PF429242 (40 *μ*M) or S1P siRNA significantly inhibited AOPP-induced sPRR generation. fPRR decoy inhibitor PRO20 and PF429242 treatment for 24 h remarkably attenuated the AOPP-induced upregulation of RAS components. Furthermore, PF429242 significantly reduced the AOPP-stimulated expression of NADPH oxidase 4 (Nox4) and H_2_O_2_ expression. The use of a small recombinant protein, named sPRR-His, reversed these alterations. In conclusion, these results provided the first demonstration of AOPP-promoted activation of sPRR. Increased renal proximal tubule Nox4-derived H_2_O_2_ contributed to the aggravation of oxidative stress. Targeting S1P-derived sPRR is a promising intervention strategy for chronic kidney disease.

## 1. Introduction

Chronic kidney disease (CKD) has emerged as a serious public health issue worldwide because of its high prevalence and high mortality associated with CKD progression and end-stage kidney disease; it is an independent risk factor for cardiovascular disease and all-cause mortality development [1, 2]. Therefore, continuing to investigate potential treatments to prevent chronic kidney injury is important.

Investigations suggested the critical role of oxidative stress in the pathogenesis, progression, and complications of CKD [3]. The NADPH oxidase (Nox) family is a major source of reactive oxygen species (ROS) [4]. The most extensively studied ROS include hydroxyl radical, superoxide anion, and H_2_O_2_ [5]. Among seven Nox families, Nox4 is the most abundant in the human kidney, predominantly in the proximal tubule [6]. The sole function of Nox4 is to produce H_2_O_2_ [7].

Advanced oxidation protein product (AOPP), formed mainly by chlorinated oxidants resulting from myeloperoxidase activity, is produced under oxidative conditions and recognized as a novel marker of oxidative damage [8]. Not only are AOPPs products of chronic oxidative stress but also they could trigger oxidative stress and further stimulate ROS generation, leading to an unstoppable positive-feedback loop [9]. AOPPs activate the intrarenal renin-angiotensin system (RAS) through mechanisms involving CD36, protein kinase C alpha, Nox, and nuclear factor-*κ*B signaling, leading to intrinsic renal cells, including endothelial cells, podocytes, and tubular epithelial cells, and excessive production of intracellular superoxide upon stimulation [10–13]. However, the molecular mechanisms underlying the pharmacological actions of AOPP remain unclear.

In 2002, Nguyen and colleagues cloned full-length (pro)renin receptor (fPRR), which directly bound to renin and prorenin preferentially, thereby triggering renal RAS [14, 15]. fPRR belongs to the type I transmembrane receptor family, characterized by containing an interesting 28 kDa soluble PRR (sPRR) and generated by site-1 protease (S1P) [16, 17]. Intensive clinical studies have proven that sPRR levels are significantly upregulated in various diseases and disease models, such as severe heart failure, hemodialysis maintenance, and primary aldosteronism, thus suggesting the value of sPRR as a biomarker [18–21]. sPRR is hypothesized to bind to specific ligands and receptors and mediate many signal transduction pathways [22–30]. For example, S1P-derived sPRR through the activation of intrarenal RAS and ENaC mediated Ang II-induced hypertension [26]. It targeted vasopressin receptor 2 to enhance urine-concentrating capability [29]. In addition, S1P-derived sPRR promoted inflammation via the Nox4/NF-*κ*B pathway and upregulated proinflammatory cytokines (IL-6 and IL-8) and adhesion molecules (vascular cell adhesion protein 1 (VCAM-1)) [31]. S1P-derived sPRR was also shown to promote fibronectin via the activation of the AKT/*β*-catenin/snail pathway in HK-2 cells [28].

This study investigated the role of S1P-derived sPRR in AOPP-elicited oxidative accentuation injury in renal tubule cells and its potential signaling pathways.

## 2. Materials and Methods

### 2.1. Reagents

*α*1-Antitrypsin Portland (*α*1-PDX) and furin inhibitor I (decanoyl-Arg-Val-Lys-Arg-chloromethylketone) were from Calbiochem. The ADAM19 inhibitor GM6001 (C_20_H_28_N_4_O_4_) was from MedChemExpress. The S1P inhibitor PF429242 (C_25_H_35_N_3_O_2_) was from AdooQ Biosciences. Human serum albumin (HSA) was from Sigma. PRO20 (L^1^PTDTASFGRILLKKMPSVR^20^) was synthesized by Shenzhen Huada Gene Co., Ltd. sPRR-His was manufactured by Qinghong Biotech.

### 2.2. Preparation of AOPP-HSA

Fatty acid-free HSA solution (30 mg/ml) was incubated with 100 mM HOCl (Fluke) at room temperature for 30 min without free amino acids, free carbohydrates, and lipids. Then, the preparation was dialyzed against phosphate buffer solution (PBS, Life Technologies) to remove free hypochlorous acid at 4°C overnight.

### 2.3. Cell Culture and Cell Culture Reagents

A human kidney proximal tubule epithelial cell line (HK-2) was purchased from Procell Life Science Co., Ltd. The HK-2 cells were cultured in Dulbecco's modified Eagle's medium/F12 medium containing 10% fetal bovine serum, penicillin (200 U/ml), and streptomycin (200 mg/ml, all obtained from Procell Life Science) at 37°C in a 5% CO_2_ atmosphere. After 12 h of serum starvation, the cells were treated with either 100 *μ*g/ml AOPP, 100 *μ*g/ml HSA, 4 *μ*M PRO20 [32], 50 *μ*M furin inhibitor I, 5 *μ*M *α*1-PDX, 25 *μ*M GM6001, 40 *μ*M PF429242, or 50 nM recombinant sPRR (sPRR-His) for 1 h, or they were transfected with either fPRR siRNA (Origene), Nox4 siRNA (Ambion), or S1P siRNA (Origene) for 24 h. These cells were then used to analyze fPRR/sPRR protein expression, while the cell medium for sPRR (Immuno-Biological Laboratories) and Ang II (Enzo Life Sciences) concentrations was analyzed by using ELISA kits in accordance with the manufacturer's instructions. Cell content was normalized to protein content and expressed as pg/mg protein.

### 2.4. Small Interfering RNA (siRNA)

RNA interference was implemented using siRNA to silence endogenous fPRR, S1P, and Nox4. HK-2 cells were grown to 70%–80% confluence in six-well culture plates and transfected with fPRR, S1P, Nox4 siRNAs, or control nontargeting siRNA. After the HK-2 cells were transfected with siRNA for 24 h with HiPerFect Transfection Reagent (Qiagen), the supernatant was removed with siRNA and AOPP was added for a further 24 h. Then, 48 h after transfection, the cells were harvested and subjected to RNA analysis. For Western blotting analysis, the HK-2 cells were transfected with siRNA for 24 h, the supernatant was removed with siRNA, medium was changed to normal growth medium for additional 24 h, and AOPP was added for a further 24 h. The transfected cells were collected 72 h after transfection with Nox4 siRNA, and the protein concentrations of sPRR and H_2_O_2_ in the medium were examined by ELISA 72 h posttransfection.

### 2.5. Measurement of TBARS and H_2_O_2_

The level of malondialdehyde (MDA) in HK-2 cells was tested using a commercially available kit (TBARS Assay Kit, Cayman Chemical Company) in accordance with the manufacturer's protocol [33]. Lysate H_2_O_2_ was measured using a ROS-Glo H_2_O_2_ Assay kit (Promega), following the manufacturer's instructions.

### 2.6. Measurement of Medium Renin Activity

At the end of treatment, cell media were harvested for subsequent analysis. Medium renin activity was defined by the concentrations of Ang I release at 37°C minus Ang I release at 4°C (Ang I Peptide Enzyme Immunoassay, Peninsula Laboratories International). The values were corrected for total protein concentration and expressed as ng/ml/h Ang I.

### 2.7. Measurement of Cell Lysate ACE Activity

Cell lysates were prepared to measure ACE activity [10]. In brief, an aliquot sample was incubated with synthetic ACE-specific substrate hippuryl-histidyl-leucine. The liberated His-Leu was converted into a fluorescent product by incubating with o-phthaldialdehyde. Then, the fluorescence of samples was detected with a MRX microplate reader (Dynex Technologies) at an emission wavelength of 495 nm upon excitation at 365 nm.

### 2.8. Western Blotting

Protein extraction and Western blotting analysis were performed as previously described [16]. Cells were washed three times with cold PBS and lysed with RIPA lysis buffer (Beyotime) with added AEBSF (Beyotime) on ice. The homogenized cells were centrifuged for 10 min at 12,000 g at 4°C. Protein samples (40 *μ*g) were mixed with 5× loading buffer (Beyotime Institute of Biotechnology). The mixture was incubated in a metal bath at 100°C for 10 min. Proteins (30 *μ*g) were resolved by SDS-polyacrylamide gel electrophoresis (SDS-PAGE) and transferred onto nitrocellulose (Amersham Pharmacia Biotech) membranes for Western blotting. After being transferred, nonspecific binding was blocked with 5% nonfat dry milk in 1× Tris-buffered saline-Tween-20 (TBST) at RT for 1 h. Subsequently, the membranes were incubated with primary antibodies (anti-PRR antibody, Sigma; anti-S1P antibody, Abcam; anti-Nox4, Abcam; and anti-*β*-actin, Beyotime) overnight at 4°C. After incubation, the membranes were washed again three times with TBST at RT (7 min per wash) and incubated with secondary antibodies (goat anti-rabbit, 1 : 2,500 dilution in TBST; goat anti-mouse 1 : 5,000 dilution in TBST; Beijing Zhongshan Jinqiao Biotechnology) for 1 h. Protein bands were visualized using the ECL detection kit (Beyotime). Densitometric analysis of each band was performed on Image-Pro Plus 6.0.

### 2.9. Quantitative Real-Time Reverse Transcription-PCR (qRT-PCR)

RNA protocols and qRT-PCR were carried out as previously described [34]. Gene expression was normalized to GAPDH. The cells were snap frozen in the TRIzol reagent (CWBio). All samples were treated with DNase digestion during RNA purification by using the RNase-Free DNase kit (Kirgen). RNA was converted to cDNA by using the High-Capacity cDNA Reverse Transcription Kit (Qiagen). The sequences of the primers (Shanghai Sangon Company) are listed in Table 1. qRT-PCR was performed using SYBR Green Master Mix (Toyobo) and a LightCycler 480 Real-Time PCR System (Roche). The PCR program was as follows: 95°C for 10 min; 40 cycles of 95°C for 5 s, 55°C for 10 s, and 72°C for 15 s; and 72°C for 7 min. All qRT-PCR reactions were carried out in duplicate.

### 2.10. Statistical Analysis

Summary data are presented as means ± SE. Student's *t*-test and the Kruskal-Wallis nonparametric statistical test followed by Dunn's multiple comparison test were performed using GraphPad Prism 6 (GraphPad Software) for statistical analysis. All data points were included in the statistical analyses. The Shapiro-Wilk test was used to confirm the Gaussian distributions of raw data. A *p* value lower than 0.05 (*p* < 0.05) was considered statistically significant.

## 3. Results

### 3.1. AOPP Induced Cleavage of fPRR, but Unmodified HSA of the Same Concentration Had No Effect in HK-2 Cells

**Figure 1:**
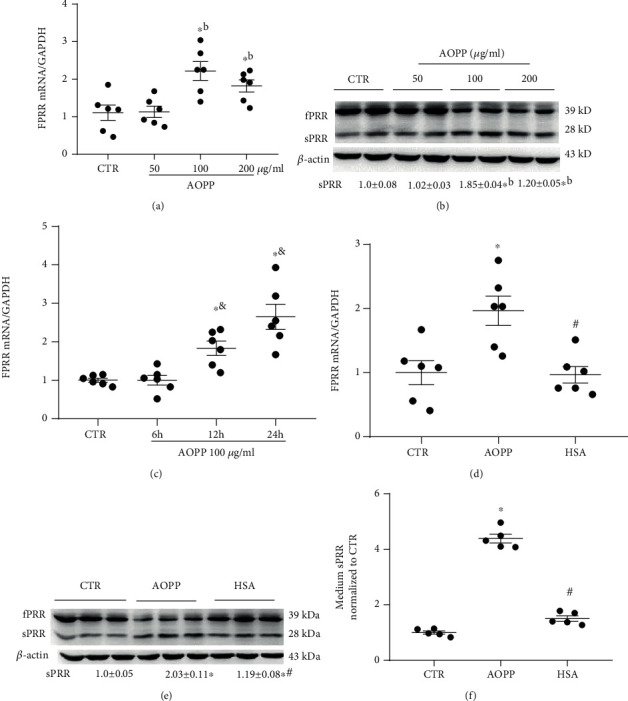
AOPP treatment activated fPRR cleavage to generate sPRR. HK-2 cells were incubated with various concentrations of AOPP (50, 100, and 200 *μ*g/ml) for 24 h, and fPRR mRNA levels were determined by qRT-PCR (a) and Western blotting (b, representative picture). With the use of AOPP (100 *μ*g/ml) for the indicated time period (6, 12, and 24 h), the expression of fPRR mRNA levels was determined by qRT-PCR (c). Values were normalized to GAPDH for qRT-PCR. *β*-Actin was used as a loading control (*n* = 6 per group). ^∗^*p* < 0.05 versus CTR; ^b^*p* < 0.05 versus 50 *μ*g/ml AOPP; and ^&^*p* < 0.05 versus 6 h AOPP. In separate experiments, HK-2 cells were treated up to 24 h with control (CTR), AOPP (100 *μ*g/ml), or unmodified HSA (100 *μ*g/ml) for 24 h. (d) qRT-PCR for fPRR mRNA expression. Values were normalized to the housekeeping gene GAPDH (*n* = 6 per group). (e) fPRR/sPRR protein expression was analyzed by Western blotting (e, representative figure) and normalized to *β*-actin (*n* = 6 per group). (f) Supernatants from cells were used for the measurement of sPRR protein by ELISA and normalized for protein content (*n* = 5 per group). ^∗^*p* < 0.05 versus CTR; ^#^*p* < 0.05 versus 100 *μ*g/ml AOPP. Data are mean ± SE; *n* is the sample size per group. All experiments were repeated independently twice with similar results, and all attempts of replication were successful.

As shown in Figure 1, HK-2 cells were cultured for 24 h with different final concentrations of AOPP-HSA (100, 200 *μ*g/ml). In the medium, the induced fPRR was cleaved to generate sPRR, as analyzed by qRT-PCR detection of the increased fPRR mRNA levels and Western blotting detection of the augmented sPRR protein expression; sPRR reached the peak at 100 *μ*g/ml AOPP (Figures 1(a) and 1(b)). In addition, AOPP-HSA (100 *μ*g/ml) upregulated fPRR mRNA expression levels in a time-dependent manner (Figure 1(c)). In order to eliminate the interference of unmodified albumin, exposure of HK-2 cells to AOPP-HSA (100 *μ*g/ml) significantly induced fPRR cleavage events, whereas HSA under the same protein concentration has had no effect. As shown in Figure 1(d), AOPP-HSA (100 *μ*g/ml) upregulated the expression of fPRR mRNA levels, whereas HSA (100 *μ*g/ml) had no effect. As shown in Figure 1(e), AOPP-HSA (100 *μ*g/ml) upregulated the expression of sPRR protein expression, whereas HSA (100 *μ*g/ml) had no effect. As shown in Figure 1(f), AOPP-HSA (100 *μ*g/ml) significantly promoted the secretion of sPRR by more than four times in the medium, whereas HSA (100 *μ*g/ml) had no effect.

These results showed that AOPP-induced fPRR cleavage produced sPRR in HK-2 cells.

### 3.2. AOPP Induced Activation of the RAS System, and Inhibition of fPRR Significantly Inhibited It

Previous literature reported that AOPP upregulated the expression of nearly all members of RAS in cultured proximal tubule epithelial cells [10]. Therefore, the role of fPRR in AOPP-induced RAS activation was examined.

**Figure 2:**
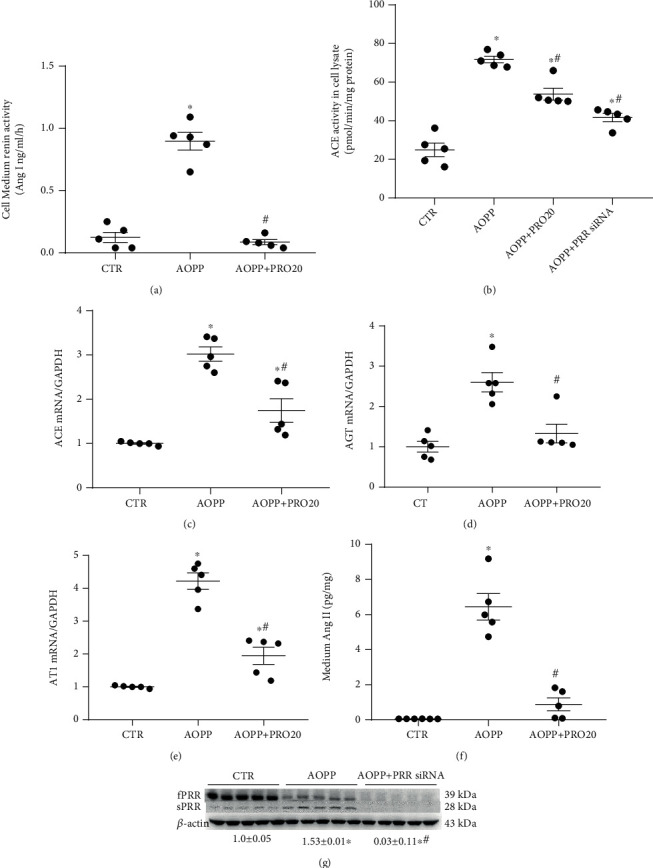
AOPP treatment-activated intrarenal RAS components were mainly mediated by fPRR in HK-2 cells. HK-2 cells were treated up to 24 h with CTR, AOPP (100 *μ*g/ml) alone, or AOPP in combination with PRO20 (4 *μ*M) or fPRR siRNA. (a) ELISA detection of cell medium renin activity. (b) ACE activity in cell lysates. (c–e) qRT-PCR analysis of ACE, AGT, and AT1R mRNA expression in cell lysates and normalization by GAPDH (*n* = 5 per group). (f) ELISA analysis of medium Ang *ΙΙ*. (g) fPRR and sPRR protein expression levels were detected by Western blotting. ^∗^*p* < 0.05 versus CTR; ^#^*p* < 0.05 versus AOPP. Data are mean ± SE; *n* is the sample size per group. In all transfection experiments, the sample CTR, AOPP alone, or AOPP with PRO20 indicated the transfected cells with control nontargeting siRNA. All experiments were repeated independently twice with similar results, and all attempts of replication were successful.

Figure 2(a) shows that the cell-medium renin activity significantly increased in the AOPP (100 *μ*g/ml) group, while it was nearly normalized in the AOPP+PRO20 group. The ACE activity in cell lysates was elevated more than 3.5-fold in the AOPP group compared with the control group (CTR), and this elevation was significantly repressed in the AOPP+PRO20 group and AOPP+fPRR siRNA group (*p* < 0.05, Figure 2(b)). Similar trends were obtained by qRT-PCR analysis of ACE, AGT, and AT1R (*p* < 0.05, Figures 2(c)–2(e)). The cell culture medium expressing Ang II was significantly higher in the AOPP group than in the CTR group (*p* < 0.05), and this expression was also blocked by fPRR siRNA treatment (*p* < 0.05, Figure 2(f)). Western blotting was then performed to test the silencing effect of siRNA on the expression levels of PRR.  Figure 2(g) shows reduced fPRR and sPRR protein expression levels following PRR siRNA treatment.

These results suggested that AOPP may lead to proximal tubule cell injury through the fPRR-mediated activation of renal RAS.

### 3.3. AOPP Induced Activation of Renal Oxidative Stress, and Inhibition of fPRR Significantly Inhibited It

**Figure 3:**
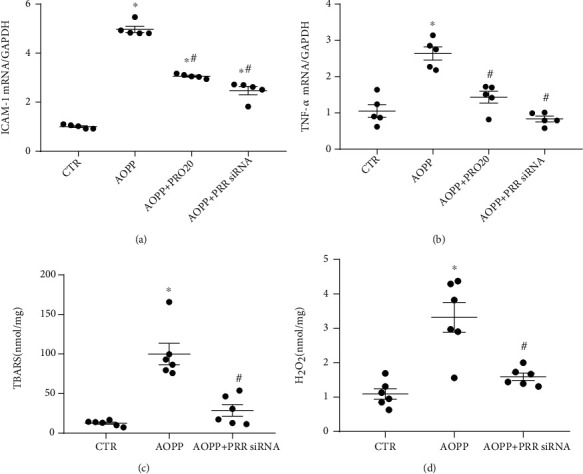
AOPP treatment-induced ICAM-1, TNF-*α*, TBARS, and H_2_O_2_ upregulated expression levels were mainly mediated by fPRR in HK-2 cells. The HK-2 cells were treated up to 24 h with CTR, AOPP (100 *μ*g/ml), AOPP+PRO20, and/or AOPP+fPRR siRNA. (a, b) qRT-PCR for ICAM-1 (a) and TNF-*α* (b) mRNA expression with normalization by GAPDH (*n* = 5 per group). (c) Cell lysate TBARS. The TBARS levels were standardized by the protein content of each sample (*n* = 6 per group). (d) Cell lysate H_2_O_2_. ELISA data of cell lysate samples normalized with protein concentrations (*n* = 6 per group). ^∗^*p* < 0.05 versus CTR; ^#^*p* < 0.05 versus AOPP. Data are mean ± SE; *n* is the sample size per group. In all transfection experiments, the sample CTR, AOPP alone, or AOPP with PRO20 indicated the transfected cells with control nontargeting siRNA. All experiments were repeated independently twice with similar results, and all attempts of replication were successful.

To the best of the authors' knowledge, compelling evidence revealed the close association between increased AOPP and oxidative stress [35]. Therefore, intercellular adhesion molecule 1 (ICAM-1), TNF-*α*, TBARS, H_2_O_2_, and the markers for oxidative stress were examined in the present study. Cell lysates' ICAM-1 and TNF-*α* mRNA levels were significantly higher in the AOPP group than in the control group, and this expression was also blocked in the AOPP+PRO20 and AOPP+fPRR siRNA groups (*p* < 0.05, Figures 3(a) and 3(b)). Similar results were acquired by ELISA analysis of TBARS and H_2_O_2_ (*p* < 0.05, Figures 3(c) and 3(d)).

### 3.4. AOPP Induced fPRR Cleavage to Produce sPRR, Which Is Mainly Mediated by S1P

**Figure 4:**
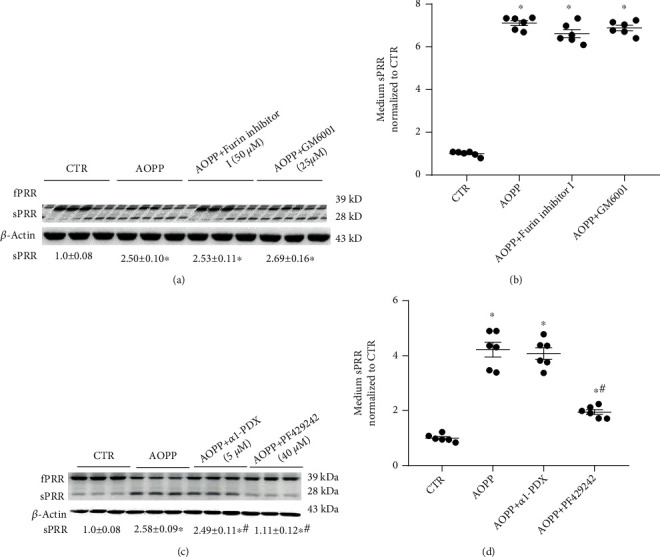
Furin inhibitor I and ADAM inhibitor GM6001 failed to affect fPRR cleavage, but the S1P inhibitor PF429242 significantly inhibited fPRR cleavage events induced by AOPP in HK-2 cells. The HK-2 cells were pretreated with furin inhibitor I (50 *μ*M), GM6001 (25 *μ*M), *α*1-PDX (5 *μ*M), or PF429242 (40 *μ*M) for 1 h and then incubated with 100 *μ*g/ml AOPP for 24 h. fPRR and sPRR protein expression levels were detected by Western blotting (a, representative picture) and normalized to *β*-actin. Densitometry analysis is shown below Western blotting (*n* = 6 per group). (b) sPRR protein concentration in culture medium was determined by ELISA, and the values were normalized to protein content (*n* = 6 per group). ^∗^*p* < 0.05 versus CTR; ^#^*p* < 0.05 versus AOPP. Data are mean ± SE; *n* is the sample size per group. All experiments were repeated independently twice with similar results, and all attempts of replication were successful.

As shown in Figures 4(a) and 4(b), furin inhibitor I could inhibit the cleavage of fPRR, but it did not affect the production of sPRR, and GM6001 completely did not affect the cleavage events of fPRR, according to Western blotting and ELISA. As shown in Figures 4(c) and 4(d), another furin inhibitor, *α*1-PDX, could also inhibit the cleavage of fPRR, but it did not affect the production of sPRR. Further, furin inhibitor I treatment-induced PRR upregulation was modest at the mRNA level compared with AOPP treatment (*p* < 0.05, Supplementary Figure 1A). This finding may explain the reason for furin inhibitors inhibiting the cleavage of fPRR. However, the S1P inhibitor PF429242 significantly inhibited the cleavage of fPRR to produce sPRR, and the contents of sPRR in the medium were significantly reduced by more than 50% compared with those in the AOPP group (*p* < 0.05).

### 3.5. AOPP Induced Activation of Renal Local RAS and Oxidative Stress, and Inhibition of S1P-Derived sPRR Significantly Inhibited This Activation

**Figure 5:**
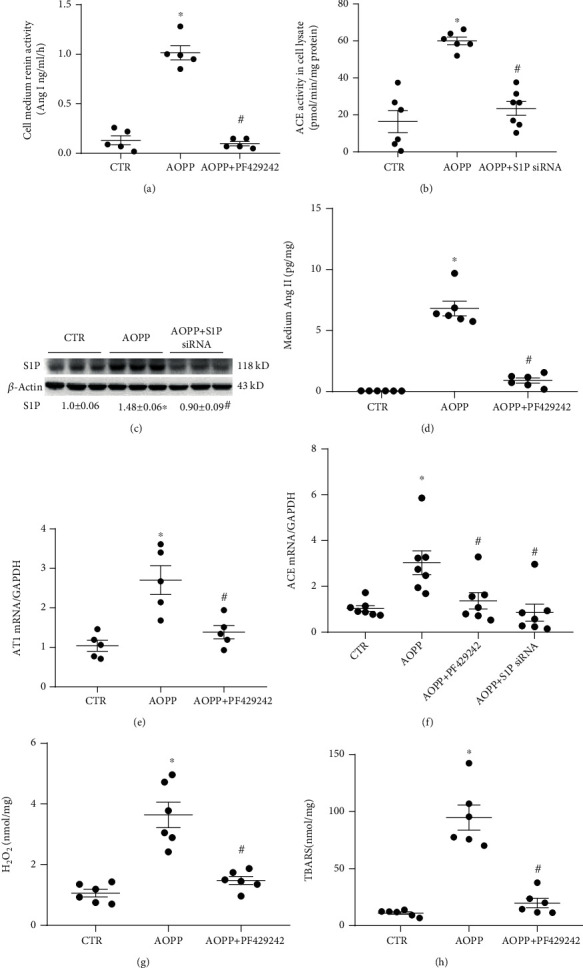
AOPP treatment-induced RAS component activation was mediated by S1P-derived sPRR in HK-2 cells. The HK-2 cells were treated up to 24 h with CTR, AOPP (100 *μ*g/ml) alone, or AOPP in combination with PF429242 (40 *μ*M) or transfected with S1P siRNA. (a) ELISA detection of cell medium renin activity. (b) ACE activity in cell lysate by the fluorometric method. (c) S1P protein expression levels were analyzed by Western blotting. (d) ELISA analysis of medium Ang II. (e, f) qRT-PCR for AT1R (e) and ACE (f) mRNA expression with normalization by GAPDH (*n* = 5 per group). (g) Cell lysate H_2_O_2_. The ELISA data of the cell lysate sample were normalized with protein concentrations. (h) Cell lysate TBARS. The TBARS levels were standardized by the protein content of each sample. ^∗^*p* < 0.05 versus CTR; ^#^*p* < 0.05 versus AOPP. Data are mean ± SE; *n* = 5–6 per group, where *n* is the sample size per group. In all transfection experiments, the sample CTR, AOPP alone, or AOPP with PF429242 indicated the transfected cells with control nontargeting siRNA. All experiments were repeated independently twice with similar results, and all attempts of replication were successful.

As shown in Figures 5(a), 5(b), and 5(d), medium renin activity, lysate ACE activity, and Ang II were all significantly higher in the AOPP group than in the CTR group (*p* < 0.05), and expression was blocked by PF429242 or S1P siRNA treatment (*p* < 0.05). The protein expression after S1P siRNA knockdown was evaluated by Western blotting analysis.  Figure 5(c) shows that S1P siRNA effectively reduced the upregulated expression levels of S1P proteins induced by AOPP.

The mRNA expression levels of AT1R and ACE in the AOPP group were upregulated compared with those in the CTR group (*p* < 0.05), and these increases were blocked by PF429242 or S1P siRNA (*p* < 0.05, Figures 5(e) and 5(f)).

Cell lysates and medium were collected and assayed for oxidative stress markers H_2_O_2_ and TBARS by using ELISA to test whether S1P-derived sPRR influenced the activation of renal oxidative stress induced by AOPP in HK-2 cells. Medium H_2_O_2_ and TBARS were higher in the AOPP group than in the CTR group (*p* < 0.05), and this expression was blocked by PF429242 (*p* < 0.05, Figures 5(g) and 5(h)).

### 3.6. AOPP Induced Activation of the Nox4/H_2_O_2_ Pathway, and Inhibition of S1P-Derived sPRR Significantly Inhibited It

ROS includes superoxide anion, H_2_O_2_, hydroxyl radical, and so on [36]. The Nox families catalyze the regulated formation of ROS [37]. Among these Noxs, we found that AOPP induced significant upregulation of Nox4 on mRNA levels but Nox1, Nox2, Nox3, and Nox5 showed no significant difference at the mRNA levels compared to the AOPP group in HK-2 cells as assessed by qRT-PCR (Supplementary Figure 2A-E). Nox4 mainly produces H_2_O_2_ rather than superoxide anions, while other Nox isoforms present in the cardiovascular system (i.e., Nox1, Nox2, Nox3, and Nox5) produce superoxide anions as their primary products [4]. In other words, the downstream expression product of Nox4 is H_2_O_2_ [38].

**Figure 6:**
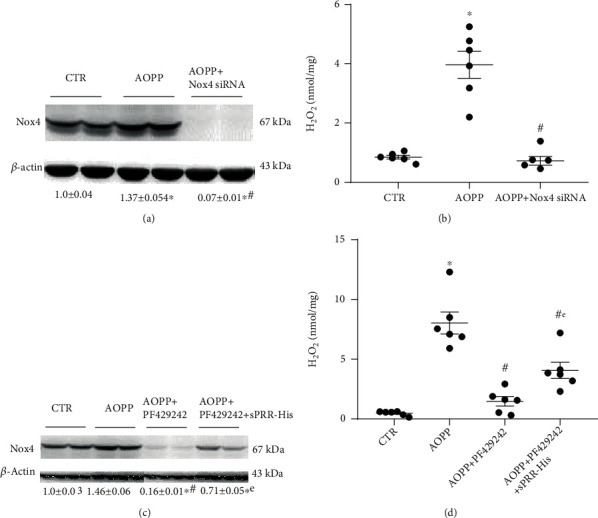
AOPP treatment-induced Nox4-dependent H_2_O_2_ production was mediated by S1P-derived sPRR in HK-2 cells. (a, b) HK-2 cells were treated up to 24 h with CTR, AOPP (100 *μ*g/ml), and AOPP+Nox4 siRNA. The expression of Nox4 was determined by Western blotting (a, representative picture) and normalized to *β*-actin. Densitometry analysis is shown below Western blotting (*n* = 6 per group). (b) The H_2_O_2_ concentrations in media were determined by ELISA and normalized based on total protein contents (*n* = 6 per group). (c ,d) HK-2 cells were incubated with PF429242 (40 *μ*M) and PF429242 (40 *μ*M)+sPRR-His (50 nM) for 1 h and then treated with AOPP (100 *μ*g/ml) for 24 h. The expression levels of Nox4 were subjected to SDS-PAGE and Western blotting (c, representative picture) and normalized to *β*-actin. Densitometry analysis is shown below Western blotting (*n* = 6 per group). (d) The contents of H_2_O_2_ in media were determined by ELISA and normalized based on total protein contents (*n* = 6 per group). ^∗^*p* < 0.05 versus CTR; ^#^*p* < 0.05 versus AOPP; and ^e^*p* < 0.05 versus AOPP+PF429242. Data are mean ± SE. *n* = 6 per group, where *n* is the sample size per group. In all transfection experiments, the samples CTR and AOPP indicated the transfected cells with control nontargeting siRNA. All experiments were repeated independently twice with similar results, and all attempts of replication were successful.

AOPP could induce the increase in Nox4 protein expression, and Nox4 siRNA completely silenced it (Figure 6(a)). Nox4 knockdown suppressed AOPP-induced Nox4 upregulation at the mRNA level (*p* < 0.05, Supplementary Figure 1B). In addition, Nox4 siRNA treatment did not affect the AOPP-induced medium sPRR secretion in HK-2 cells (*p* > 0.05, Supplementary Figure 1C). Therefore, Nox4 may be a downstream target of PRR/sPRR signaling.

AOPP-induced cell lysate H_2_O_2_ significantly increased, and this increase was blocked by PF429242 treatment (*p* < 0.05, Figure 6(b)). As shown in Figure 6(c), PF429242 almost completely blunted the AOPP-induced upregulated expression of Nox4 (*p* < 0.05, Figure 6(b)), and an exogenous recombinant protein-sPRR-His rescued this phenomenon. Similar results were obtained by ELISA analysis of H_2_O_2_ (*p* < 0.05, Figure 6(c)). AOPP induced the increase in cell lysate H_2_O_2_ expression, which was significantly inhibited by PF429242 and further reversed by sPRR-His (*p* < 0.05, Figure 6(d)).

## 4. Discussion

AOPP was discovered by Witko-Sarsat et al. in 1996 in the plasma of patients who developed chronic renal failure [39]. It is one of the specific markers of protein oxidation, and it is related to the damage of oxygen free radicals in the body and oxidative stress reaction [40]. AOPP is a product of oxidative stress, and it may induce or aggravate oxidative stress response and chronic inflammation [41]. It is an independent predictor of renal injury in patients with CKD [42]. The level of AOPP was positively correlated with the degree of renal function injury [43, 44]. In addition, a growing body of evidence suggested that AOPP could promote the progression of CKD and induce the apoptosis of podocytes, damage of renal tubular epithelium, and proliferation and differentiation of renal mesangial cells [12, 45–47]. Studies suggested that AOPP could induce the rapid generation of oxygen free radicals' (0^2−^) renal epithelial cell line, leading to the activation of Nox, which is similar to phagocytes and mainly derived from tubular epithelial cells, and the increase in ROS production [48]. A previous study well demonstrated that AOPP contributed to the intrarenal RAS activation involvement of CD36-dependent signaling [10]. However, whether AOPP could activate fPRR and the extracellular domain of soluble PRR-sPRR, a new member of RAS, is unclear. In the current study, AOPP induced fPRR to be cleaved by S1P to generate sPRR, which then activated intrarenal RAS. Meanwhile, sPRR mediated oxidative stress response by affecting the Nox4/H_2_O_2_ pathway. Moreover, we also found that Nox4 siRNA treatment did not affect AOPP-induced medium sPRR secretion in HK-2 cells. Therefore, we speculated that other pathways irrelevant to oxidative stress should be included in the secretion of sPRR. Indeed, antioxidant treatment was not sufficient to halt the vicious circle of cause and causality in renal injury. The phenomenon was similar to that found in a previous study [49]. In the Heart Outcomes Prevention Evaluation (HOPE) study, vitamin E (an antioxidant) administered daily for four to six years had no beneficial effects on cardiovascular outcomes in patients at high risk for cardiovascular events.

RAS plays an important role in the occurrence and progression of nephropathy [50]. In particular, clinical use of ACEI and ARB could effectively control blood pressure and reduce renal injury, such as proteinuria [51]. fPRR is highly expressed in renal tubules, and it has a potential role in renal RAS regulation [52]. Many studies have shown that fPRR could aggravate tissue damage, such as promoting the development of hypertension, diabetic nephropathy, and proteinuria nephropathy, by activating local RAS in tissues and activating the role of proinflammatory and profibrosis factors [34, 53–56]. The data in the present study showed that fPRR inhibitors PRO20 and fPRR siRNA could significantly inhibit the AOPP-induced expression levels of AGT, ACE, AT1R, and Ang II in HK-2 cells. Therefore, these novel findings may provide a new therapeutic target for early intervention in the treatment of CKD. Conceivably, the AOPP-related oxidative stress and inflammation to activate RAS and Nox4/H_2_O_2_ signaling may be a new therapeutic target for renal intervention in CKD.

Evidence from literature suggested that furin or ADAM19 cleaved fPRR to sPRR, but the phenomenon remains controversial [57, 58]. Recent studies have well identified that a novel proprotein convertase, called S1P, is the primary protease responsible for cleaving and producing sPRR [16, 17]. In the present study, the S1P inhibitor PF429242 significantly reduced the production of sPRR induced by AOPP. Moreover, two furin inhibitors effectively inhibited fPRR cleavage, but they did not affect sPRR secretion. We also found that mRNA levels of PRR were modestly elevated in treatment with furin inhibitor I compared to AOPP treatment. Such interesting phenomenon needs further investigation. We speculated that a novel sPRR that has not yet been detected by Western blotting and ELISA may be present, which could be cleaved by furin. While we did not have direct evidence, we suspected that furin inhibition reduced the production of the novel sPRR, which further elicited the upregulation of PRR mRNA levels. Overall, these findings extended the previous observations and supported the notion that S1P is the major protease responsible for the cleavage and production of sPRR. Previous studies showed that sPRR promoted inflammation via the Nox4/NF-*κ*B pathway and upregulated IL-6, IL-8, and VCAM-1 [31]. Another study highlighted the role of sPRR in renal fibrosis, where sPRR was shown to promote fibronectin in HK-2 cells via the activation of the AKT/*β*-catenin/snail pathway [28]. The present study revealed that AOPP could activate not only renal RAS but also the Nox4/H_2_O_2_ pathway. Inhibition of sPRR could not only reduce the expression levels of renin, Ang II, AGT, ACE, and AT1R but also directly regulate the expression of Nox4 and further affect the production of H_2_O_2_ in HK-2 cells. Meanwhile, sPRR-His rescued the expression of Nox4 and the production of H_2_O_2_. These results suggested that sPRR could affect the oxidative stress injury of proximal tubule epithelial cells through RAS-dependent and RAS-independent pathways.

Oxidative stress is one of the factors that cause chronic vascular disease [59]. As an important subtype of NADPH oxidase, Nox4 is highly expressed in the kidney, and it could catalyze the production of H_2_O_2_ [60]. PRR regulates the stimulating activation of *α*-ENaC by regulating Nox4-mediated H_2_O_2_ production [61]. In the present study, sPRR-His and the S1P inhibitor PF429242 were used to investigate the role of sPRR in Nox4 regulation by AOPP. The results showed that AOPP-induced Nox4 expression was significantly inhibited after inhibiting sPRR production, while exogenous sPRR-His recombinant protein could upregulate Nox4 expression. Based on these results, sPRR-His may be involved in regulating the expression of Nox4. However, the specific molecular mechanism of Nox4 by sPRR is still missing, which could be explored in future experiments.

Taken together, this study demonstrated that the sPRR in renal tubular epithelial cells plays an important physiological and pathophysiological role in renal oxidative stress and inflammation through activating intrarenal RAS and Nox4/H_2_O_2_ signaling induced by AOPP.

## Figures and Tables

**Table 1:** Primer sequences for qRT-PCR analysis.

Primer name	Primer sequence
GAPDH F	GGAGCGAGATCCCTCCAAAAT
GAPDH R	GGCTGTTGTCATACTTCTCATGG
AGT F	ACAATGAGAGTACCTGTGAGCA
AGT R	TCTTGGCCTGAATTGGAGCAG
AT1R F	ATTTAGCACTGGCTGACTTATGC
AT1R R	CAGCGGTATTCCATAGCTGTG
ACE F	GGAGGAATATGACCGGACATCC
ACE R	TGGTTGGCTATTTGCATGTTCTT
S1P F	ACCTCGAAACAATCCATCCAGT
S1P R	ACTTGAGGGAACGAAAGACTTTT
ICAM-1 F	ATGCCCAGACATCTGTGTCC
ICAM-1 R	GGGGTCTCTATGCCCAACAA
TNF-*α* F	CCTCTCTCTAATCAGCCCTCTG
TNF-*α* R	GAGGACCTGGGAGTAGATGAG
Nox-1 F	TTGTTTGGTTAGGGCTGAATGT
Nox-1 R	GCCAATGTTGACCCAAGGATTTT
Nox-2 F	ACCGGGTTTATGATATTCCACCT
Nox-2 R	GATTTCGACAGACTGGCAAGA
Nox-3 F	ACCGTGGAGGAGGCAATTAGA
Nox-3 R	TGGTTGCATTAACAGCTATCCC
Nox-4 F	CAGATGTTGGGGCTAGGATTG
Nox-4 R	GAGTGTTCGGCACATGGGTA
Nox-5 F	ACTCAGCAGTTTAAGACCATTGC
Nox-5 R	GGACTCTTTCACATGCAGAGC
fPRR F	CAGACGTGGCTGCATTGTCC
fPRR R	CTGGGGGTAGAGCCAGTTTGTT

## Data Availability

No data were used to support this study.

## Supplementary Materials

Supplementary 1 Supplementary Figure 1: (A) HK-2 cells were treated up to 24 h with CTR, AOPP (100 *μ*g/ml), and AOPP+furin inhibitor I. (B and C) HK-2 cells were treated up to 24 h with CTR, AOPP (100 *μ*g/ml), and AOPP+Nox4 siRNA. (B) The level of Nox4 mRNA was determined by qRT-PCR and normalized to GAPDH. (C) Medium sPRR. ELISA data normalized with protein concentrations (*n* = 6 per group). ^∗^*p* < 0.05 versus CTR; ^#^*p* < 0.05 versus AOPP. Data are mean ± SE. *n* = 6 per group, where *n* is the sample size per group.

Supplementary 2 Supplementary Figure 2: HK-2 cells were treated up to 24 h with CTR and AOPP (100 *μ*g/ml). The levels of Nox1, Nox2, Nox3, Nox4, and Nox5 mRNA were determined by qRT-PCR and normalized to GAPDH. (A) Nox1 mRNA, (B) Nox2 mRNA, (C) Nox3 mRNA, (D) Nox4 mRNA, and (E) Nox5 mRNA. ^∗^*p* < 0.05 versus CTR. n.s.: nonsignificant. Data are mean ± SE. *n* = 6 per group, where *n* is the sample size per group.
