# Supplementary figures and images for: Advanced Oxidation Protein Product Promotes Oxidative Accentuation in Renal Epithelial Cells via the Soluble (Pro)renin Receptor-Mediated Intrarenal Renin-Angiotensin System and Nox4-H2O2 Signaling
Source: Oxid Med Cell Longev. 2021 Nov 26;2021:5710440. doi: 10.1155/2021/5710440 (PMC8642821; doi:10.1155/2021/5710440)

**A**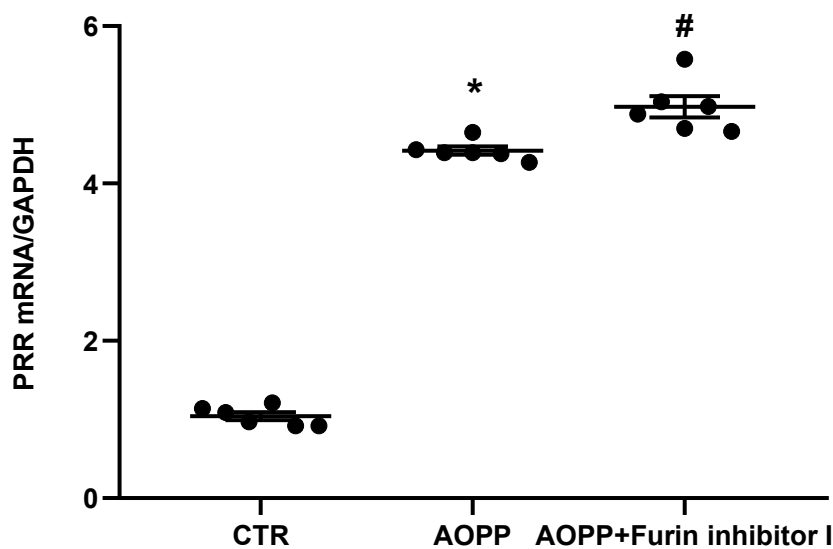**B**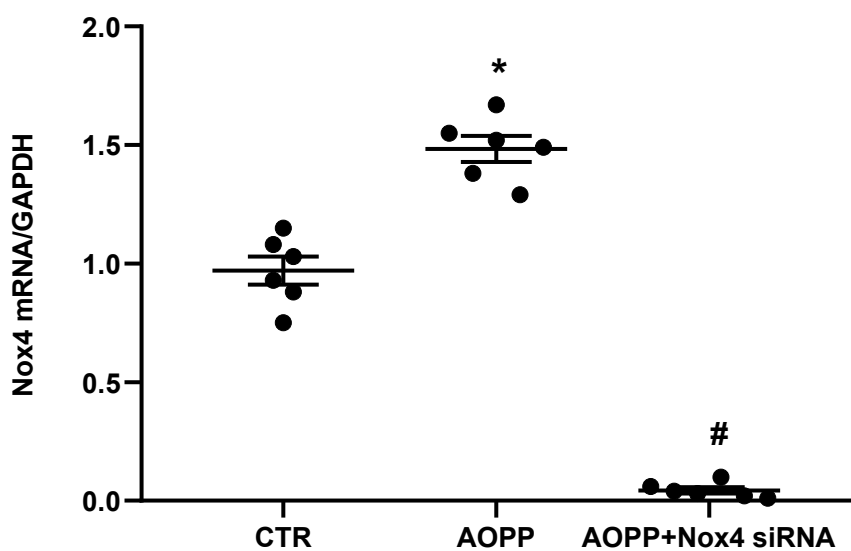**C**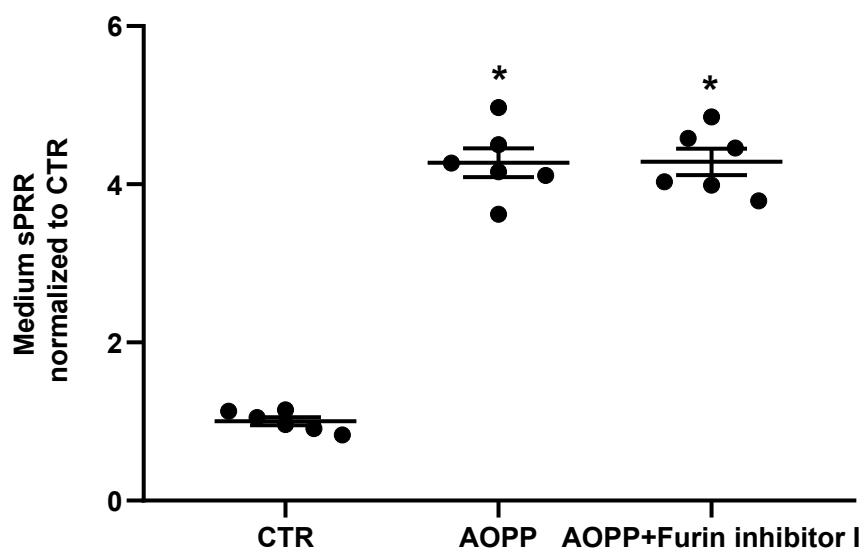

Supplement: Supplementary 1 — Supplementary Figure 1: (A) HK-2 cells were treated up to 24 h with CTR, AOPP (100 μg/ml), and AOPP+furin inhibitor I. (B and C) HK-2 cells were treated up to 24 h with CTR, AOPP (100 μg/ml), and AOPP+Nox4 siRNA. (B) The level of Nox4 mRNA was determined by qRT-PCR and normalized to GAPDH. (C) Medium sPRR. ELISA data normalized with protein concentrations (n = 6 per group). ∗p < 0.05 versus CTR; #p < 0.05 versus AOPP. Data are mean ± SE. n = 6 per group, where n is the sample size per group. [file 5710440.f1.pdf]

**A**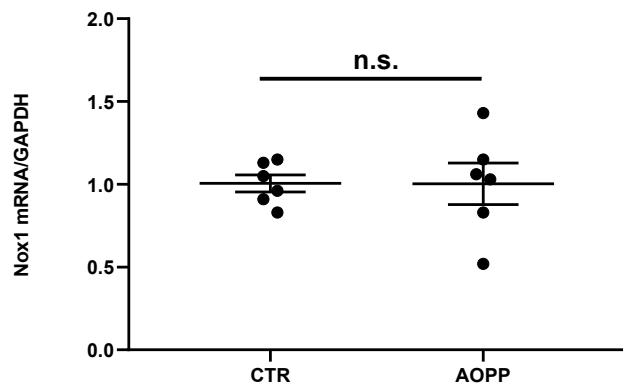**B**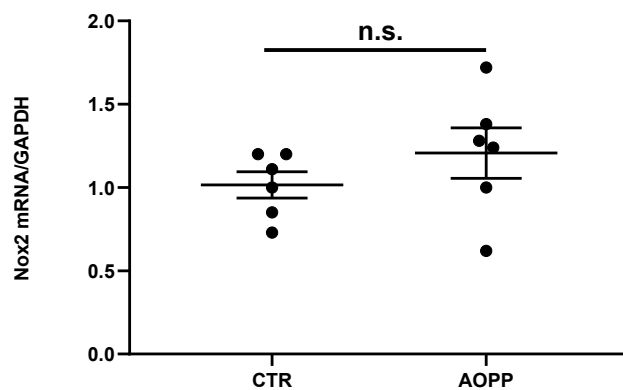**C**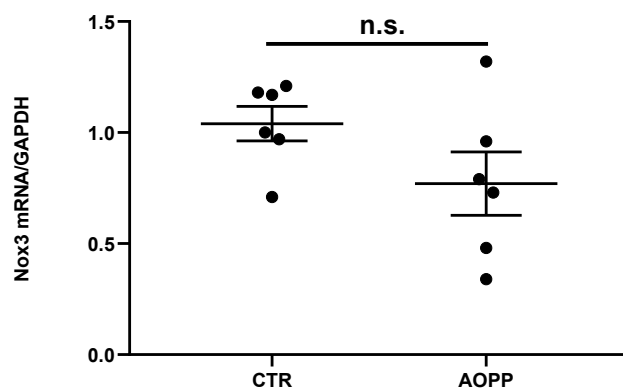**D**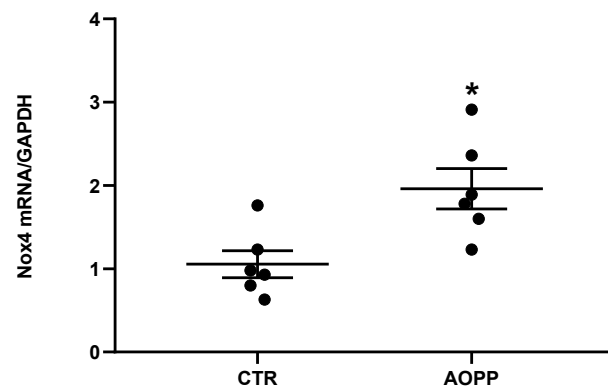**E**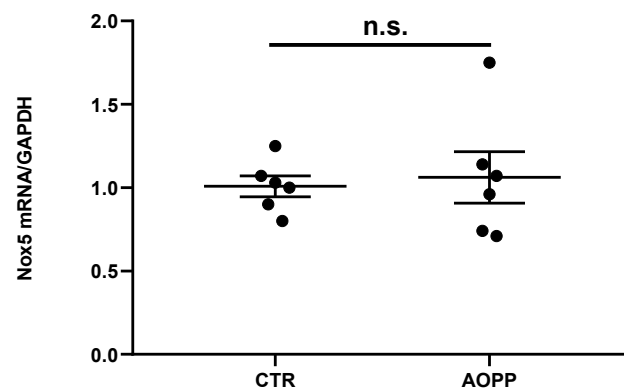

Supplement: Supplementary 2 — Supplementary Figure 2: HK-2 cells were treated up to 24 h with CTR and AOPP (100 μg/ml). The levels of Nox1, Nox2, Nox3, Nox4, and Nox5 mRNA were determined by qRT-PCR and normalized to GAPDH. (A) Nox1 mRNA, (B) Nox2 mRNA, (C) Nox3 mRNA, (D) Nox4 mRNA, and (E) Nox5 mRNA. ∗p < 0.05 versus CTR. n.s.: nonsignificant. Data are mean ± SE. n = 6 per group, where n is the sample size per group. [file 5710440.f2.pdf]
